# Balancing the benefits of vaccination: An *envy-free* strategy

**DOI:** 10.1093/pnasnexus/pgae087

**Published:** 2024-02-26

**Authors:** Pedro Ribeiro de Almeida, Vitor Hirata Sanches, Carla Goldman

**Affiliations:** Instituto de Física, Departamento de Física Geral—Universidade de São Paulo, São Paulo-SP 05508-090, Brasil; Instituto de Física, Departamento de Física Geral—Universidade de São Paulo, São Paulo-SP 05508-090, Brasil; Instituto de Física, Departamento de Física Geral—Universidade de São Paulo, São Paulo-SP 05508-090, Brasil

**Keywords:** pandemic preparedness, balanced vaccine allocation, decision making process, envy-free division, direct and indirect benefits

## Abstract

The Covid-19 pandemic revealed the difficulties of vaccinating a population under the circumstances marked by urgency and limited availability of doses while balancing benefits associated with distinct guidelines satisfying specific ethical criteria. We offer a vaccination strategy that may be useful in this regard. It relies on the mathematical concept of envy-freeness. We consider finding balance by allocating the resource among individuals that seem heterogeneous concerning the direct and indirect benefits of vaccination, depending on age. The proposed strategy adapts a constructive approach in the literature based on Sperner’s Lemma to point out an approximate division of doses guaranteeing that both benefits are optimized each time a batch becomes available. Applications using data about population age distributions from diverse countries suggest that, among other features, this strategy maintains the desired balance, throughout the entire vaccination period. We discuss complementary aspects of the method in the context of epidemiological models of age-stratified Susceptible - Infected - Recovered (SIR) type.

**Significance Statement**

The share with which a single dose contributes to the direct and indirect effects of vaccination may depend, among other conditions, on the age of the individual who receives it. This imposes difficulties in optimizing allocation guidelines that aim to support individual needs while controlling transmissibility. A strategy of vaccination based on the mathematical concept of envy-freeness offered in the present study reveals a tendency to balance the benefits of vaccination locally, within a country, and among countries presenting the most diverse age-distribution profiles. Results obtained for the fractions of the doses allocated for each age group that keep the balance across time should allow for an interplay with age-stratified SIR-type models revealing complementary aspects of the two models.

## Introduction

The unprecedented situation in which a vaccine was successfully developed amid the disease pandemic, the case of Covid-19, brought about urgent questions related to the possible strategies for allocation of the doses made available gradually in very small batches that do not cover the entire population in a community all at once (1, 2). Given the high transmissibility of the virus and widespread infection, the problems associated with vaccine allocation highlighted the urgent need to elaborate and put into practice certain guidelines that best satisfy a given set of ethical requirements (3, 4). In many countries, the prioritization followed protocols suggested by the WHO (1) to allocate the first doses that became available to the oldest and to those with comorbidity since these individuals are the most likely to develop severe forms of the disease (5, 6). Other groups at maximum risk as health care workers and, in some places, members of disadvantaged groups deprived of minimal protection against direct exposure to the virus, the homeless for instance, in addition to members of indigenous populations and isolated small communities, among others, have also been eligible for doses of the vaccine from the first batches (1).

In reality, after the most elderly and other most at-risk groups receive their doses, virtually the entire population remains unvaccinated, while new doses continue to become available in limited numbers. We restrict the contribution offered here to this scenario. In the remaining susceptible individuals, comprising the large majority of the population, the ability to transmit the disease and the severity of the symptoms are widely dispersed, and these generally correlate with age.

The elderly within this remaining population would still be mostly benefited directly from receiving the doses because they tend to develop the most severe forms of the disease compared to the younger that are likely to present with only mild symptoms, although this is not a rule (5). On the other hand, due to their mobility and intense daily activity, younger people have a major capacity to transmit the virus compared to that of the elderly (3). Therefore, vaccinating younger people would greatly benefit the entire population, as an indirect effect.

Direct and indirect effects of vaccination, regarding mainly the interplay between decreasing disease severity and its transmissibility, raise questions about the possibility of balancing these two aspects that seem to compete with each other in making decisions about allocating vaccine doses (3). We believe that any solution to this problem should comprise the following points: (i) a measure to evaluate proximity to a balanced condition that enables comparing results among different strategies of vaccination and (ii) a methodology to implement dose allocation that maintains such balance on time until vaccine coverage of the entire population is achieved.

We argue that this can be approached following an *envy-free* type of strategy for a fair division of doses among individuals possessing different utilities. The concept of *envy-free*ness is often illustrated in the literature by the classical cake-cutting problem (7, 8). This refers to a partition among agents of a certain resource (the cake), generally heterogeneous, such that each one of the agents evaluates their parts as being the best among the parts chosen by the others. The heterogeneity of the resource is usually expressed through a utility function that assigns to the different parts of the cake, different values satisfying additivity. In general, each agent has its own utility function. Here, we use the notion of utility to quantify both the direct and indirect benefits of vaccinating the diverse age groups of the population. We then formulate such a strategy to address the case of vaccine dose allocation by the agents to the individuals adapting the constructive approach presented in Ref. (9) and reviewed in Ref. (10) which is based on Sperner’s Lemma. For this, we assume that the vaccine is a desirable good and also that individuals and vaccine doses can be conceived as divisible quantities being represented by densities, defined appropriately. Accordingly, each age group receives a score named *utility* in agreement with the various views and plans of certain consultants or *counselors* expressing their priority criteria in line with the current public policies in the considered community.

We examine the case at issue regarding transmissibility and severity of the disease as a prototype to explain our ideas, although the model is not restricted to it. In keeping with this, it will be sufficient to consider the expertise of only two counselors, each one in charge of scoring all individuals according to their ages. One of the counselors referred to as $C_{A}$ (Ana), is an expert in predicting the ways of spreading the disease. The other counselor, $C_{B}$ (Bob), is an expert in disease symptomatology. Specifically, $C_{A}$ represents the allocation guideline that accounts for the benefit of vaccinating to control transmission—the indirect effect of vaccination. $C_{B}$ represents another allocation guideline that accounts for the benefit at the individual level—the direct effect of decreasing the severity of the symptoms. Both $C_{A}$ and $C_{B}$ are interested in balancing the two aspects; none of them wants to dispute vaccine doses. Therefore, whenever a new batch becomes available to this community, the doses will be allocated in such a way that each counselor agrees on the distinct groups of individuals to be vaccinated to optimize separately the benefit envisaged by each one. We claim that this characterizes an *envy-free* division of the vaccine doses.

The way that this may be accomplished is our main proposal and will be developed in the following sections. We emphasize that unlike *utilitarian models* (11, 12), our strategy is not based on a single-score system for which the priorities for doses allocation are evaluated in terms of the total score received by each individual from the different counselors. Rather, we conceive the model in such a way that each counselor optimizes the benefit according to their particular view. Our approach differs also from the *reserve system* strategies (13) for which the total vaccine supply from each batch is distributed according to preassigned proportions to certain reserved categories. In our model, the proportions of doses attributed to the management of each counselor are dynamic quantities, resolved along the process.

Our proposal is presented in the Model section. Illustrative examples are considered in the Results section. We compare the results from the application of the *envy-free* strategy using data comprised of certain population age distributions, with those predicted by the other two strategies examined, referred to as *oldest-first,* and *maximize-benefit*, as detailed below. We have also considered a strategy named *minimize-benefit* to set a scale to measure the efficiency with which the benefits are acquired by each strategy. These comparative results indicate that the *envy-free* leads to a considerable improvement in keeping the benefits related to $C_{A}$ and $C_{B}$ close together over time conferring support to this strategy as a way to pursue the desired balance. Other vaccination schemes have been considered in the literature in connection with the time evolution of age-stratified epidemiological models of Susceptible - Infected - Recovered (SIR) type, as in Refs. (14–17). We found it interesting then to discuss in the Discussion and conclusion section the extent of the scheme proposed in connection to possible interactions with SIR-type models. In the supplementary material, we outline the numerical code (18) prepared to implement the model dynamically, automating the whole procedure, except for the prioritization of the different age groups, which remain under the charge of the counselors.

## Model

Our formulation is decomposed into two parts. The first part consists of preliminary definitions to build up the relevant simplex as a basis for the choice of individuals to be vaccinated at each time. It is assembled using accessible data about population age distribution, taken in connection with the utility attributed to all groups of individuals by each counselor. The second part consists in building up the dynamics that drives this choice to achieve the required balance between the two guidelines.

### Outline: the cake-cutting analogy

Our construction is founded in the classic example of settling a type of fair division of a cake—a desirable good—to be distributed between two individuals expressing distinct preferences for its different regions whether for being creamier or for being filled with more chocolate or for any other reason that distinguishes preferences (10). To think of the cake mathematically, it is helpful to project it into a line. Each point of this line divides the cake into two parts allowing both individuals to figure out the one they prefer, depending upon their individual tastes for the regions embraced. At a point of this line defined as an *envy-free* point, the individuals express preferences for distinct parts of the cake such that neither envies the other’s choice. Our approach to distributing vaccines is based on finding an *envy-free* point defined accordingly to divide the age-ordered population (the analogous of the cake) into two parts. The allocation of doses between these two parts removes the corresponding number of individuals (which, following the analogy, diminishes the cake) to optimize the benefits of both counselors. However, there are crucial differences to implement the method. One difference is that the batches do not cover the entire population all at once. Therefore, the equivalent procedure to the envy-free division of the cake shall be repeated several times until vaccination coverage is achieved. This introduces an additional dynamic process—a repeating procedure—to account for allocating each new batch that arrives, in general, with a variable number of doses supposedly being fully allocated within the time interval before the arrival of the next batch. The other crucial difference between the two cases is that both counselors benefit from each single susceptible who is vaccinated. In the case of the cake, each person benefits exclusively from the part they receive. Our proposal intends to incorporate these differences into the cake-cutting idea. The following description shows how we solve the part of the dynamics corresponding to finding an approximate envy-free division of a given number of doses *V* composing a single batch, at a time *t*. The repeating procedure is explained in the supplementary material.

### The simplex

The age group distribution in a community with *N* susceptible individuals will be considered for the account of two counselors $C_{A}$ and $C_{B},$ each one of them endowed with a utility *function*  $g_{\eta }(I),$  η=A, B hypothesized to attribute a score to each individual according to the corresponding age-related priority criteria. This can be performed by ordering these *N* individuals in a way that their age I(x) is a monotonic increasing surjective function of their positions x∈Z∩[0,N].

To build up the *1-simplex* of interest over which we perform our considerations about the choices of the counselors, we map the interval [0,N] into the interval [0,1] and variable *x* into a real variable y=x/N such that y∈[0,1]. The age at position *y* within this map shall now be calculated as I(yN). We may assume that all individuals within the same age group are equally valued by each counselor though most likely, the value varies between counselors. It will be convenient though, to deal with continuous utility functions $g_{\eta }(I(yN))\equiv \rho_{\eta }(y)$ represented by a combination of smoothed step functions for each counselor η=A, B, as detailed in the supplementary material. The *utility densities*  $u_{\eta }(y)$ defined for all y∈[0,1] as


(1) $$u_{\eta }(y)=\frac{\rho_{\eta }(y)}{\int_{0}^{1}\rho_{\eta }(y))\;dy}$$

are the functions that allow for considerations about *envy-free* divisions based on Sperner’s Lemma, as will be explained next. Observe also that any region *ω* of the considered simplex may be decomposed into a number *M* of disjoint subregions $\{v_{\;j}\},$  j=1,2,…,M. Each $v_{\;j},$ extended between endpoints $y_{\;j\text{I}}$ (initial) and $y_{\;j\text{F}}$ (final) with $y_{\;j\text{I}}<y_{\;j\text{F}}$, comprises a number $\left[(y_{\;j\text{F}}-y_{\;j\text{I}})N\right]$ of individuals, where the notation [z] indicates the integer part of a real number *z*.

### The dynamic

Consider a population that at a certain instant of time *t* comprises N(t) susceptible individuals to whom a batch of V<N(t) vaccine doses shall be allocated. We suppose that the availability of the batches occurs at a certain frequency of 1/T until the entire population is vaccinated. We also assume that individuals achieve full protection after receiving a single dose. The time *t* shall then be better measured in terms of the interval *T* between batches as t=nT, for $n\in Z_{+}$. The question posed here regards the choice of the *V* individuals to receive the doses at each *t* to balance the current guidelines.

We think of two different priority criteria suggested by two *counselors*  $C_{A}$ and $C_{B}$ expressing different opinions about how one should rank the population in the community to guide this choice. The *utility density functions*  $u_{A}(y)$ and $u_{B}(y),$  y∈[0,1] [1] conceived, respectively, by $C_{A}$ and $C_{B}$ assume nonnegative real values and represent a measure of the relevance for vaccinating the individuals ordered according to some rule. To present the methodology, we choose to order the individuals by their age, although this does not exclude any other possibility. Such an ordered list of individuals mapped into the interval [0,1] defines the *1-simplex* (see The simplex section). We aim to present a fair division strategy of an *envy-free* class through which the choice of the *V* individuals at each time balances the perspectives of the two counselors in the best possible way. The proposal offers an approximate solution extending the constructive approach (8) based on Sperner’s Lemma as presented in Ref. (9) and reviewed in Ref. (10).

From $u_{\eta }(y)$, we define the *benefit*  $U_{\eta }^{\omega (t)},\eta \in \{A,B\}$ according to the referred counselor perspective, that results from vaccinating the individuals inside a region ω(t) of the simplex at time *t*:


(2) $$U_{\eta }^{\omega (t)}\equiv \;\int_{\omega (t)}u_{\eta }(y)\;dy.$$

The total benefit that is reached after vaccinating the entire population of susceptible amounts to 1, according to both counselors:


(3) $$U_{\eta }\;=\int_{0}^{1}u_{\eta }(y)\;dy=1.$$

**Fig. 1.**
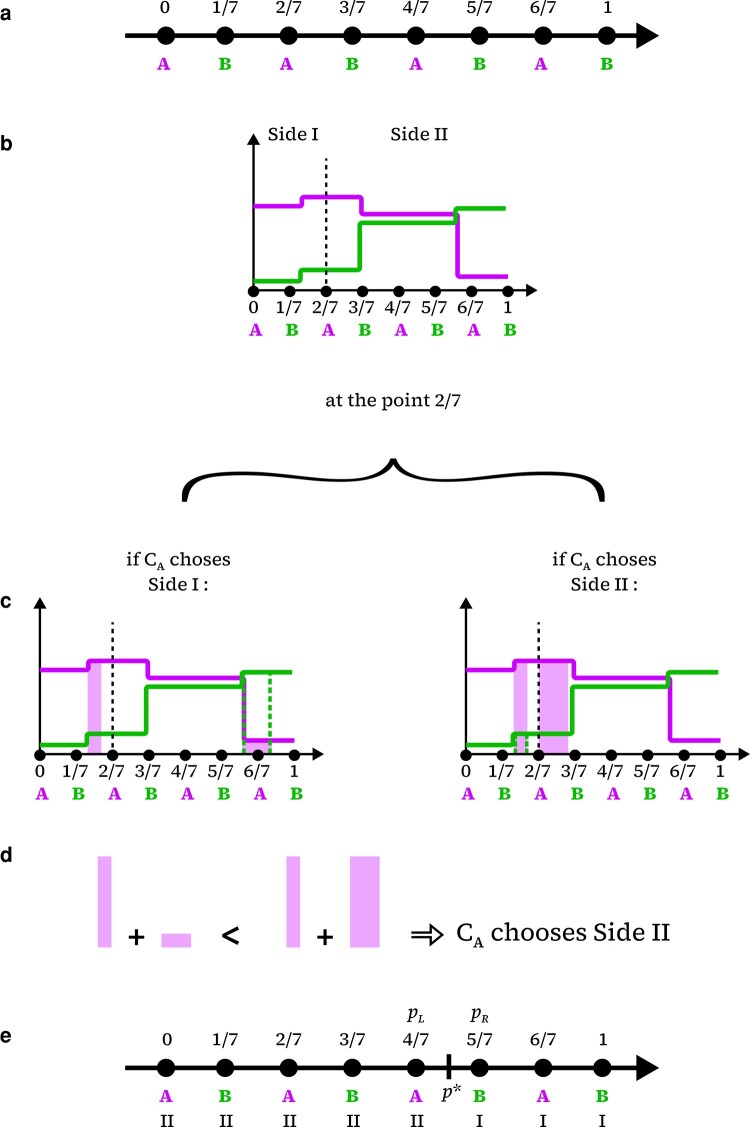
a) A defined partitioned simplex with d=7. The scheme is adapted from Ref. (10). Counselor $C_{A}$ chooses a side at all points A and counselor $C_{B}$ chooses a side at all points B. b) The traces of utility functions ( $u_{A}$ purple, $u_{B}$ green) are shown to illustrate the choice of $C_{A}$, at an arbitrary point $p_{i}=2/7$. c) Benefit evaluation on each side as the areas below the curves. d) Comparing benefits. e) Labeled simplex after both counselors, at each corresponding point, experience (b)–(d) and express their preferences for vaccinating on side I or on side II. In this example, the region shown defined by endpoints $\left(p_{L},p_{R}\right)$ encloses a point $p^{*}$ that sets an envy-free division of the simplex.

We proceed by partitioning the 1-simplex, at a time t, into a number *d* of identical parts, each of which comprised between a pair of neighbor points $\left(p_{i},p_{i+1}\right)$ at the positions $\left(y_{\;p_{i}},y_{\;p_{i+1}}\right)$, respectively, with $y_{\;p_{\;j}}=j/d,$ for j={0,1,2,…,d}. We then assign to the endpoint $p_{0}$ at $y_{\;p_{0}}=0$ a label arbitrarily chosen between A and B so called in reference to the counselors, and then proceed by labeling each of the subsequent points $p_{i}$ of the sequence as A or B, alternately (Fig. 1a). In the limit of very large *d*, as any interval $\left[y_{\;p_{i}},y_{\;p_{i+1}}\right]$ shortens, such alternation is then crucial for finding an envy-free point in between $p_{i}$ and $p_{i+1}$, as detailed next.

Notice that each point $p_{i}$ at $y_{\;p_{i}}$ splits the ordered population into two parts, part I on the left of $p_{i}$ and part II on the right of $p_{i}$, comprising, respectively, $N_{I}^{i}$ and $N_{\mathrm{II}}^{i}$ individuals at time *t*, such that


(4) $$\begin{array}{cc} & N_{I}^{i}=\left[y_{\;p_{i}}N\right],\\ & N_{\mathrm{II}}^{i}=\left[(1-y_{\;p_{i}})N\right]. \end{array}$$

At each of these points $p_{i}$, we also consider splitting the batch of vaccines available at a certain time into two parts, $V_{I}^{i}$ and $V_{\mathrm{II}}^{i}$. We choose $V_{I}^{i}$ and $V_{\mathrm{II}}^{i}$ proportional to $N_{I}^{i}$ and $N_{\mathrm{II}}^{i},$ respectively:


(5) $$\begin{array}{rl} & V_{I}^{i}=\left[y_{\;p_{i}}V\right],\\ & \;\;V_{\mathrm{II}}^{i}=\left[(1-y_{\;p_{i}})V\right]. \end{array}$$

To the extent that the simplex is arranged in this way, both quantities, i.e. individuals and vaccine doses, are evaluated using the single continuous variable *y*. This allows each counselor to express, at the corresponding labeled points $p_{i}$, what would be their preferential side to proceed with vaccination. We assume that $V_{I}^{i}$ and $V_{\mathrm{II}}^{i}$ are intended necessarily to vaccinate individuals, respectively, on sides I and II defined for each *i*. Explicitly, to maximize benefit at each A-labeled point $p_{i}$, counselor $C_{A}$ is asked to express her preference, based on $u_{A}$, about vaccinating $V_{I}^{i}$ individuals on the side I or $V_{\mathrm{II}}^{i}$ individuals on the side II. The same for counselor $C_{B}$ at each B-labeled point $p_{i},$ based on $u_{B}$. One should notice that it is implicit in this procedure, regardless of the counselors’ choices, that neither of them would be able to vaccinate the entire population at once with the corresponding amount $V_{I}^{i}$ or $V_{\mathrm{II}}^{i}$ of doses available on each side. Moreover, at an A-labeled point $p_{i}$ where counselor $C_{A}$ is in charge of choosing the side and decides for say, side II, she is supposed to make use of all of the $V_{\mathrm{II}}^{i}$ doses preassigned to that side. This implies that counselor $C_{B}$ would necessarily vaccinate individuals on the other side using all of the $V_{I}^{i}$ doses, even though it may not be his preferential choice. Despite this, $C_{B}$ would look for individuals amounting to $V_{I}^{i}$ that are best to be vaccinated on the side I according to his utility density function. The same will be followed by counselor $C_{A}$ after $C_{B}$ has expressed his preferential choices at each B-labeled point $p_{i}$.

Accordingly, the counselor in charge at each point $p_{i}$, regardless of being labeled A or B, ends up vaccinating exclusively at one of the sides. Nonetheless, they would benefit from vaccination on both sides. Since both utility density functions assume nonzero values along the entire simplex, each counselor must account for a *benefit coupled* with the other’s choice. In the example above, we understand that, at the point $p_{i},$ counselor $C_{A}$ has chosen side II and the best subregion to vaccinate the $V_{\mathrm{II}}^{i}$ individuals at that side. This choice is foreseen after evaluating the total benefit from $u_{A}$ composed of (i) the amount obtained from $u_{A}$ at a subregion of II comprising $V_{\mathrm{II}}^{i}$ individuals that have been chosen according to her utility function $u_{A}$ and (ii) the *coupled benefit* that corresponds to the amount obtained from $u_{A}$ at a subregion of I comprising $V_{I}^{i}$ individuals that have been chosen by suggestion of $C_{B},$ based on his utility density $u_{B},$ Fig. 1b and c sketches this evaluation of the benefits by $C_{A}$ at an arbitrary labeled point $p_{i}=2/7$ for illustrative utility functions, as shown. Then $C_{A}$ concluded that the sum of (i) and (ii) is greater than the amount she would have obtained if she has chosen to vaccinate on the side I and received the coupled benefit from side II (Fig. 1d). For this, one must assume that both counselors know each other’s utility density functions.

To extend (i) and (ii) to arbitrary choices, it will be useful to define for each Γ∈{I,II} and η∈{A,B}, the interval $\Omega_{\eta }^{\Gamma }(p_{i},t),$ as the subregion on the side *Γ* of the simplex with respect to a point $p_{i}$ where counselor *η* evaluates the maximum benefit from $u_{\eta }$ at time *t*. According to this, counselor $C_{A}$ looks for the largest between the *total benefit*  $U_{A}^{I}(p_{i})$ and $U_{A}^{\mathrm{II}}(p_{i})$ that, recalling 2, are defined as


(6) $$U_{A}^{I}(p_{i},\;t)\equiv U_{A}^{\Omega_{A}^{I}}+U_{A}^{\Omega_{B}^{\mathrm{II}}}$$

and


(7) $$U_{A}^{\mathrm{II}}(\;p_{i},t)\equiv U_{A}^{\Omega_{A}^{\mathrm{II}}}+U_{A}^{\Omega_{B}^{I}}$$

If $U_{A}^{I}(p_{i},t)⩾U_{A}^{\mathrm{II}}(p_{i},t)$, she decides for side I, otherwise she decides for side II.

Likewise, to decide which side to vaccinate at each B-labeled point $p_{i}$, according to his utility function, counselor $C_{B}$ looks for the largest between the *total benefit*  $U_{B}^{I}(p_{i},t)$ and $U_{B}^{\mathrm{II}}(p_{i},t),$ defined as


(8) $$U_{B}^{I}(\;p_{i},t)\equiv U_{B}^{\Omega_{B}^{I}}+U_{B}^{\Omega_{A}^{\mathrm{II}}}$$

and


(9) $$U_{B}^{\mathrm{II}}(\;p_{i},t)\equiv U_{B}^{\Omega_{B}^{\mathrm{II}}}+U_{B}^{\Omega_{A}^{I}}.$$

If $U_{B}^{\mathrm{II}}(p_{i},t)⩾U_{B}^{I}(p_{i},t)$, he decides for the side II, otherwise he decides for the side I.

The example discussed above, illustrated in Fig. 1, corresponds to the case for which the pre-evaluation of the benefit by $C_{A},$ at the considered point $p_{i},$ resulted $U_{A}^{\mathrm{II}}(p_{i},t)>U_{A}^{I}(p_{i},t)$.

Finally, after the two counselors have expressed their preferential sides at each of the corresponding points $p_{i}$ and the simplex becomes indexed analogously to the example in Fig. 1e, it allows through simple visual inspection, to list all pairs of consecutive points, referred generically as $(p_{L},p_{R}),$ such that the counselor at the point on the left $p_{L}$ has expressed a preference to vaccinate on one of the sides say on side II, while the counselor at the point on the right $p_{R}$ has expressed a preference to vaccinate on the opposite side, i.e. side I.

The existence of at least one such pair of points is ensured by Sperner’s Lemma. Accordingly, for a sufficiently large partition *d*, an internal point $p^{*}$ of the interval defined by any of these pairs will approximate a position at which the preferred sides of the two counselors are opposite to one another. The choice of any of those points $p^{*}$ (if more than one) identifying opposite preferred sides to allocate the available vaccine doses characterizes an approximate *envy-free* division at a given time *t* for which either


(10) $$U_{A}^{I}(p^{*},t)⩾U_{A}^{\mathrm{II}}(p^{*},t)\;\text{and}\;U_{B}^{\mathrm{II}}(p^{*},t)⩾U_{B}^{I}(p^{*},t)$$

or


(11) $$U_{A}^{I}(p^{*},t)⩽U_{A}^{\mathrm{II}}(p^{*},t)\;\text{and}\;U_{B}^{\mathrm{II}}(p^{*},t)⩽U_{B}^{I}(p^{*},t).$$

In order to carry on this strategy until all susceptible individuals in the population have the opportunity to get their doses, it is assumed that the procedure described above is repeated at each time *t* when a new batch containing *V* doses becomes available. For simplicity, we consider the unrealistic case for which *V* does not change along the entire process. On each of these occasions, the simplex must be rescaled and the utility densities attributed accordingly to the set of individuals mapped again into the interval [0,1], after excluding those already vaccinated with the doses from the previous batch. A schematic view of this repeating procedure is provided in the supplementary material.

We present a numerical study using this procedure for analyzing the time evolution of the benefit in selected population age distributions. The utility functions are written using an arbitrary scale to mimic counselors’ general guidance. The results are compared with those produced by strategies specified in the following as *maximize-benefit*, *oldest-first* in addition to a *minimize-benefit* strategy introduced to set a scale for efficiency. The *maximize-benefit* strategy looks for distributing the doses to the groups of individuals for which the sum of the two utilities is maximized. The *minimize-benefit* strategy looks for distributing the doses to the groups of individuals for which the sum of the two utilities is minimized. Under the *oldest-first* strategy, the doses available are fully distributed to the oldest individuals present at the time, approaching the current procedure adopted by many public health systems after the most elderly and other most at-risk groups received their doses. Our findings are shown in the next section.

### Verifying conditions for Sperner’s Lemma

Observe that even though side I has no individuals to be vaccinated at the endpoint $p_{0}$ at $y_{\;p_{0}}=0,$ the counselor in charge there might have two options: either to let the other vaccinate on side II using the entire amount *V* of doses, or to vaccinate the *V* individuals on side II. If the point $p_{0}$ is A-labeled then counselor $C_{A}$ will still be in charge to decide about her preferential side based on the largest between


(12) $$\begin{array}{cc} & U_{A}^{I}(p_{0},t)=U_{A}^{\Omega_{B}^{\mathrm{II}}(p_{0},t)}\\ & \text{and}\\ & U_{A}^{\mathrm{II}}(p_{0},t)=U_{A}^{\Omega_{A}^{\mathrm{II}}(p_{0},t)}. \end{array}$$

On the contrary, if the endpoint $p_{0}$ is B-labeled then counselor $C_{B}$ would select the side based on the largest between


(13) $$\begin{array}{cc} & U_{B}^{I}(p_{0},t)=U_{B}^{\Omega_{A}^{\mathrm{II}}(p_{0},t)}\\ & \text{and}\\ & U_{B}^{\mathrm{II}}(p_{0},t)=U_{B}^{\Omega_{B}^{\mathrm{II}}(p_{0},t)}. \end{array}$$

Since at $p_{0}$ the values reached by $u_{A}$ at $\Omega_{A}^{\mathrm{II}}(p_{0},t)$ are higher than or at least equal to the values reached by $u_{A}$ at $\Omega_{B}^{\mathrm{II}}(p_{0},t)$ then $U_{A}^{\mathrm{II}}≧U_{A}^{I}$. Similarly, since the values reached by $u_{B}$ at $\Omega_{B}^{\mathrm{II}}(p_{0},t)$ are higher than or at least equal to the values reached by $u_{B}$ at $\Omega_{A}^{\mathrm{II}}(p_{0},t)$ then $U_{B}^{\mathrm{II}}≧U_{B}^{I}$. Therefore, the counselor in charge at $p_{0}$ will oneself prefer to indicate the individuals to be vaccinated, and this would happen on side II, instead of leaving vaccination up to the other counselor. Using similar arguments, one finds that for $p_{1}$ at $y_{\;p_{1}}=1$ either one of the counselors would choose side I. Therefore, whoever counselor at $p_{0}$, would choose the right side, whereas whoever counselor at $p_{1}$, would choose the left side. These conclusions assure that the conditions under which Sperner’s Lemma holds are fully satisfied by the simplex defined above.

## Results

The time evolution of benefits acquired by applying different strategies of vaccination, including the *envy-free*, is studied through numerical simulations. The methodology outlined (supplementary material) has been developed specifically to accomplish this (18). We use data from the population age distribution of the countries indicated in Ref. (19). For comparing the outcomes from the diverse strategies, the population of each country is divided into four age groups $I_{k},$  k=1,2,3,4, comprising, respectively, individuals from 0 to 14 years old (y.o.) $\left(I_{1}\right)$, from 15 to 24 y.o. $\left(I_{2}\right)$, from 25 to 64 y.o. $\left(I_{3}\right)$, and those who are 65 y.o. or above $\left(I_{4}\right)$. Any other division could have been considered. Before proceeding into the normalization, each counselor $C_{\eta }$ assigns each group a *utility* value, or *score*, according to their particular priority criteria. We consider four scenarios named (1) Default, (2) Symptomatology, (3) Transmissibility, and (4) Concentrated, indicated in Table 1. In scenario (1), both counselors attribute the same values for their priority groups, the same values for their second groups, etc. In scenario (2), the value that $C_{B}$ (symptoms) attributes to his priority group $\left(I_{4}\right)$ (elderly), is greater than the value that $C_{A}$ attributes to her priority group $\left(I_{2}\right)$. In scenario (3), the value that $C_{A}$ (transmission) attributes to her priority group $\left(I_{2}\right)$ (working young ), is greater than the value that $C_{B}$ attributes to his priority group $\left(I_{4}\right)$. Scenario (4) is analogous to scenario (1), with larger differences between the priority groups of each counselor with respect to the other groups.

**Table 1.** The set of utilities used in the examples.

		0–14 y.o.	15–24 y.o.	25–64 y.o.	65 + y.o.
(1) Default	$C_{A}$	12	16	4	1
	$C_{B}$	1	4	12	16
(2) Symptomatology	$C_{A}$	12	16	4	1
	$C_{B}$	1	2	7	23
(3) Transmissibility	$C_{A}$	7	23	2	1
	$C_{B}$	1	4	12	16
(4) Concentrated	$C_{A}$	7	23	2	1
	$C_{B}$	1	2	7	23

**Fig. 2.**
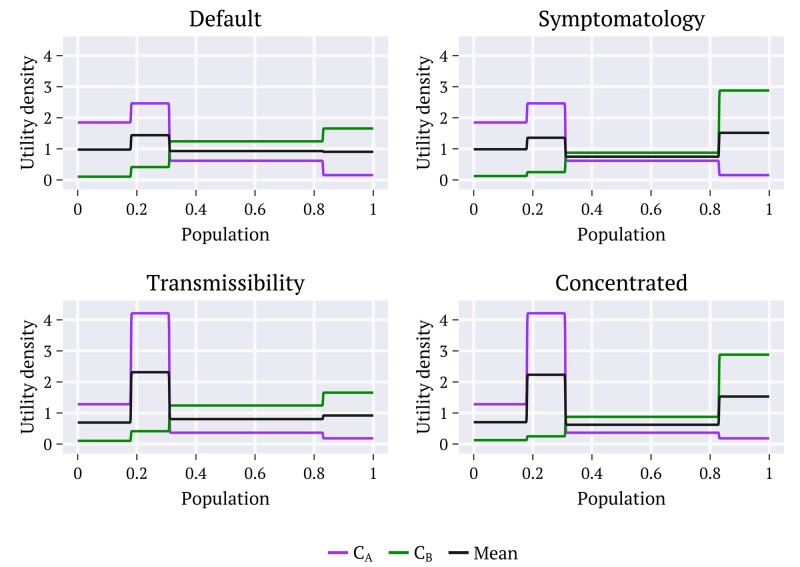
Illustrative example. Utility density functions $u_{\eta }(y)$, η=A,B evaluated from Eq. 1 for counselors $C_{A}$ (purple) and $C_{B}$ (green), respectively, with the continuous functions $\rho_{\eta }(y)$ (supplementary material) built up using the four combinations of utilities in Table 1, for the population age groups of the United States.

These are all nonzero positive values conceived using an arbitrary scale and are assumed independent of the number of individuals in each age group, characteristic of each community. These are then applied on Eq. 1 to build the utility density functions $u_{\eta }(y)$ for the population age distributions from diverse countries. The idea is to test the choices of the counselors as the utilities become concentrated on the groups that each one of them judges as the most priority to compare with the cases for which the utilities are less concentrated in a single group (Default). As illustration, the utility density functions obtained using the population age distribution of the United States are shown in Fig. 2. Analogous results have been obtained for the remaining distributions (not shown). We emphasize that the assignments above for scenarios (1)–(4) represent possible choices to compare the achievements of the different strategies and utilities considering the diverse age distributions. Any other possibility would be feasible, depending on the interests and attributions of the counselors.

Given the utilities $u_{\eta }(y)$ for each country and each counselor, the results of the simulated dynamic allow one to compare the outcomes from four different strategies to allocate doses: *envy-free*, *oldest-first, maximize-benefit,* and *minimize-benefit,* one at a time, until vaccination ends. In addition, for comparison purposes, we examine the results from a *random procedure* under which the fraction v(t) of doses is offered to a randomly chosen fraction of the simplex.

Under *envy-free*, the preferred side of each of the two counselors at the point $p^{*}(t)$ is opposite to one another. Yet, because for a given η,  $U_{\eta }^{\Gamma }(p^{*})$ accounts also for the coupled benefits associated with the other’s choice [6–9], the total region $\Omega (t{)}_{\mathrm{EF}}$ to be vaccinated at each time *t* is necessarily composed of two or more disjoint segments spanned on both sides . That is, $\Omega (t{)}_{\mathrm{EF}}=\Omega_{A}^{I}\cup \Omega_{B}^{\mathrm{II}}$ if $C_{A}$ preferred side I and $C_{B}$ preferred side II, or $\Omega (t{)}_{\mathrm{EF}}=\Omega_{A}^{\mathrm{II}}\cup \Omega_{B}^{I}$ if $C_{A}$ preferred side II and $C_{B}$ preferred side I.

Under *oldest-first*, the focus is on the protection of the elderly. In this case, the choice of the fraction of individuals to be vaccinated with available doses is based exclusively on the distribution of the age groups. The preference is always for the *V* most elderly which fraction v(t)=V/N(t) comprises a one-segment region $\Omega (t{)}_{\mathrm{oldest}}$ of the simplex at each time *t*. The *maximize-benefit* strategy, on the other hand, is based on the choice of the fraction v(t) of individuals comprising a region $\Omega (t{)}_{\max}$, eventually composed of disjoint regions, where the benefit achieved by adding the utilities from the two counselors is maximized. Analogously, the *minimize-benefit* strategy accounts for the region $\Omega (t{)}_{\min}$ where the total benefit is minimized.

Using definition 2, we express the average increment to the benefit achieved at time *t* as


(14) $$U^{\Omega (t)}=\frac{1}{2}(U_{A}^{\Omega (t)}+U_{B}^{\Omega (t)})$$

for all strategies, such that $\Omega (t)\in \{\Omega (t{)}_{\mathrm{EF}},\Omega (t{)}_{\mathrm{oldest}},\Omega (t{)}_{\max},$  $\Omega (t{)}_{\min},\Omega (t{)}_{\mathrm{rand}}\}$. These include the region $\Omega (t{)}_{\mathrm{rand}}$ achieved by the considered random procedure.

**Fig. 3.**
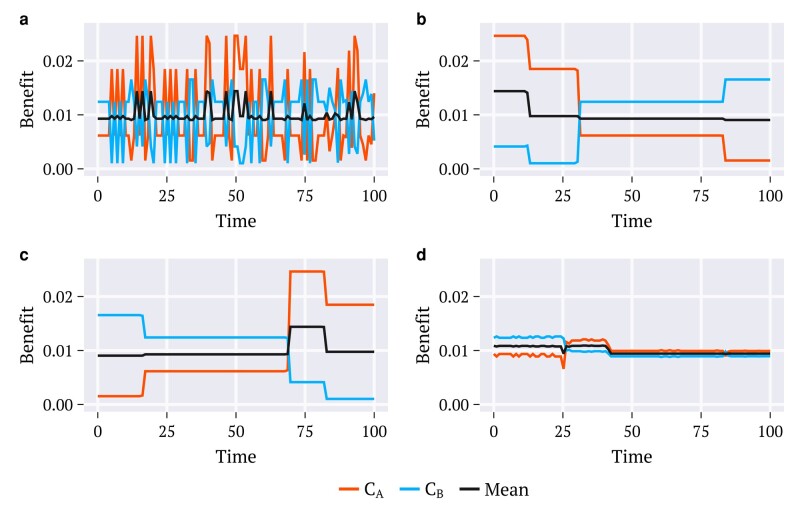
Simulated time series for the benefits [2] acquired by each counselor, $C_{A}$ (orange) and $C_{B}$ (blue) through a) a random procedure and strategies, b) *maximize-benefit*, c) *oldest-first*, d) *envy-free*. The utility density functions employed correspond to the combination defined as Default in Fig. 2 and the results shown are for the population age distribution of the United States.

**Fig. 4.**
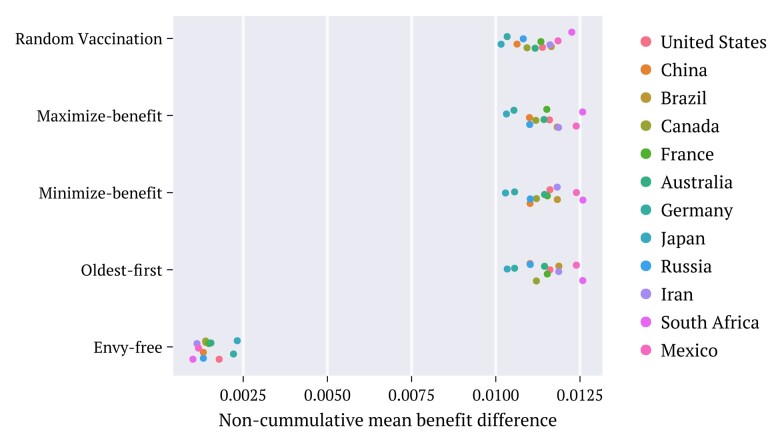
Time average of the differences (absolute values) [15] between the contributions of the two counselors to the benefits obtained through each strategy, extended to the population age groups of the selected countries, as specified. The utility density functions employed correspond to the combination defined as Default.

**Fig. 5.**
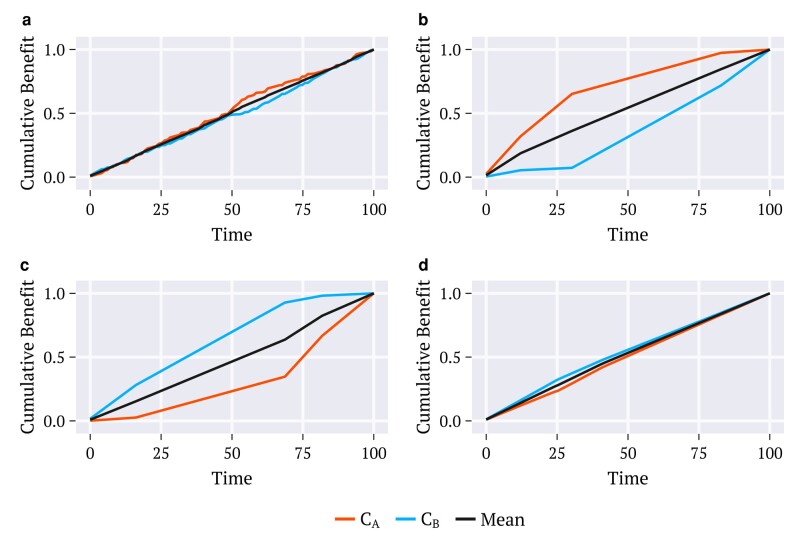
Simulated time series for the cumulative benefits $\Phi_{\eta }(t)$ [16] acquired by each counselor, $C_{A}$ (orange) and $C_{B}$ (blue) through a) a random procedure and strategies, b) *maximize-benefit*, c) *oldest-first*, d) *envy-free*. The utility density functions employed correspond to the combination defined as Default in Fig. 2. The results shown are for the population age distribution of the United States.

**Fig. 6.**
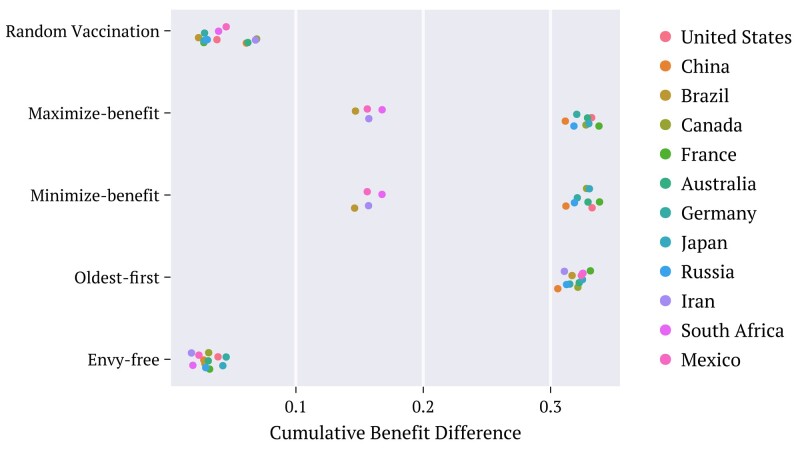
Time average of the differences (absolute values) $\bar{\Delta \Phi }$ [18] between the contributions of the two counselors to the cumulative benefits obtained through each strategy, extended to the population age distributions of the selected countries, as specified. The utility density functions employed correspond to the combination defined as Default.

**Fig. 7.**
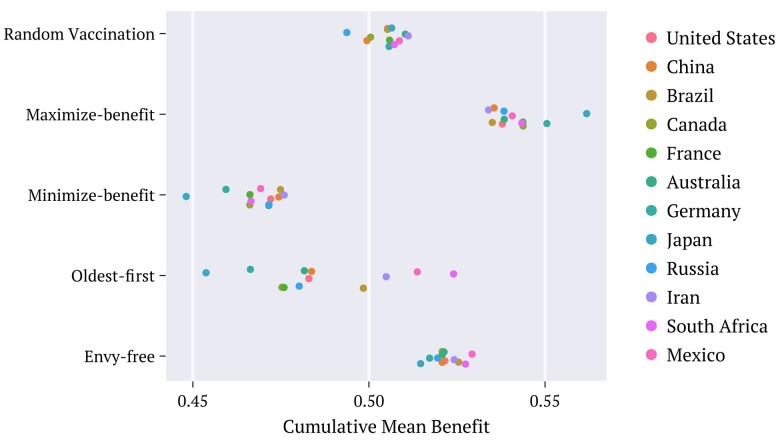
Time average of the mean cumulative benefit $\bar{\Phi }$ [19] between the contributions of the two counselors, for each strategy and selected countries. The utility density functions employed correspond to the combination defined as Default.

**Fig. 8.**
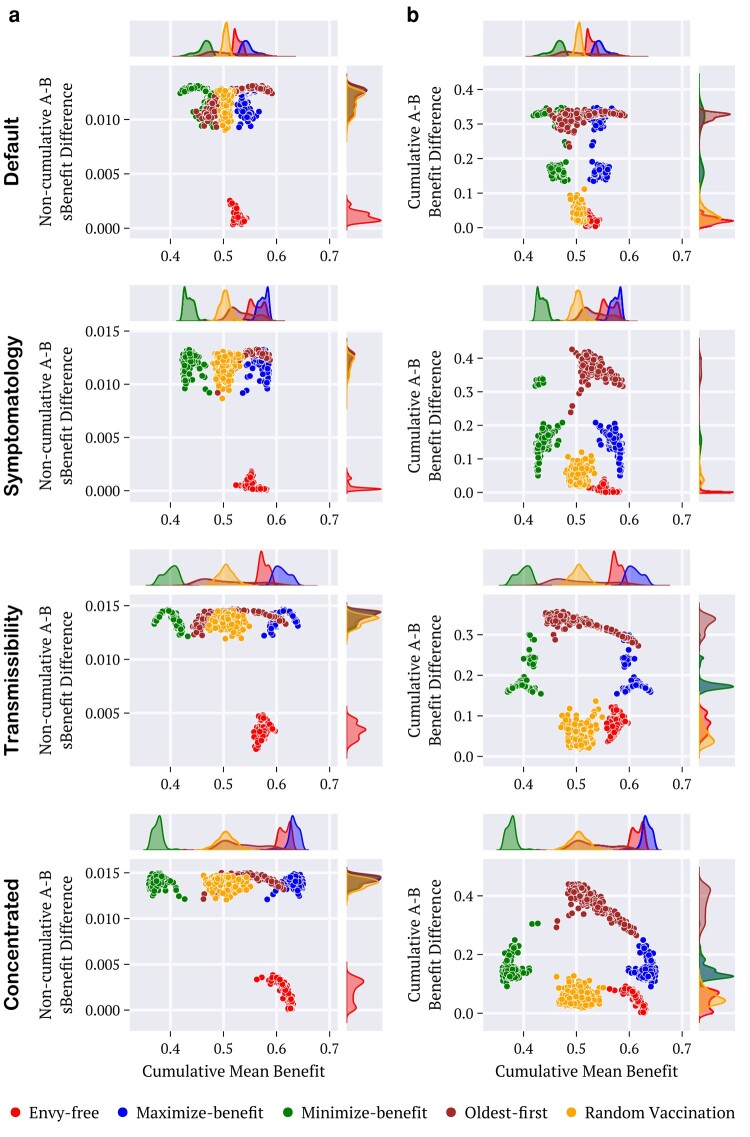
Time averages of a) the cumulative differences (absolute values) $\bar{\Delta \Phi }$ [18], and b) the instantaneous differences (absolute values) $\bar{\Delta U}$ [15] against the mean $\bar{\Phi }$ [19], evaluated for the population age distributions of 236 selected countries for strategies and utility combinations, as indicated by colors. The distributions of the points are indicated by the lateral diagrams. *Maximize-benefit* (blue) *minimize-benefit* (green), *oldest-first* (brown), *envy-free* (red), and random procedure (orange).

**Fig. 9.**
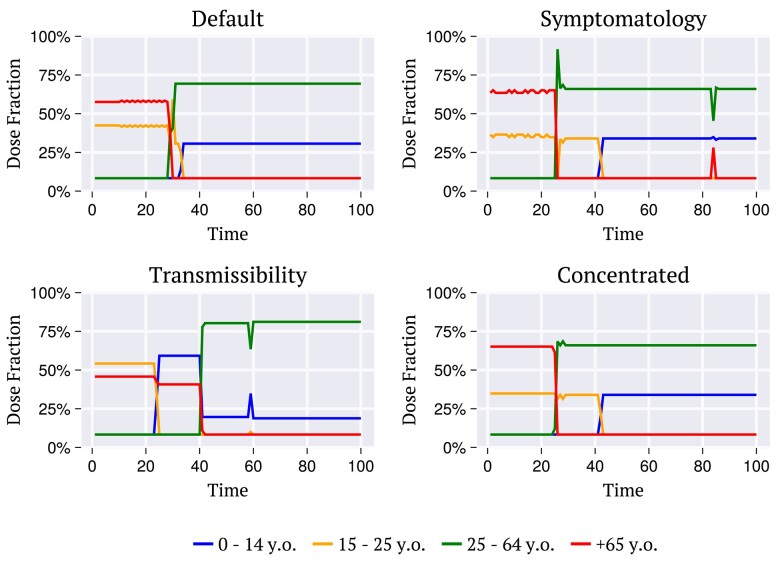
The fraction of the doses to be allocated to each age group, at each time, to follow the *envy-free* strategy considering the different combinations of utilities, as indicated in Table 1. The results shown are for the population age distribution of the United States.

In all cases, the simulations run for an initial population comprising $N(0)=10^{4}$ individuals and fixed vaccine batches of $V=10^{2}$ doses each. The simplex built at every iteration time to follow the *envy-free* strategy is partitioned using d=100 that ensures convergence of the results, as suggested by the study depicted in Fig. S2. The whole procedure intends to find the regions Ω(t) to distribute the doses at each iteration time, that conform with each of the considered strategies. Figures 3–7 show results considering utilities combined as Default (Table 1). The other combinations are also examined and the results are collected in Figs. 8 and 9.

The time behavior of the increments $U_{A}^{\Omega (t)},U_{B}^{\Omega (t)}$, and $U^{\Omega (t)}$ in Fig. 3 are for the population of the United States. All other distributions examined exhibit the same patterns (results not shown). These results certify that under the envy-free strategy, the increments to the benefit acquired at each time by each counselor remain very close together until vaccination is completed. Such behavior contrasts with those obtained through oldest-first and maximize-benefit for which the increments to the two benefits differ considerably from each other across time. By adopting any of these two strategies, the selected regions of the simplex for doses allocation, either $\Omega =\Omega_{\mathrm{oldest}}$ or $\Omega =\Omega_{\max}$ along which the utility densities $u_{A}(y)$ and $u_{B}(y)$ may assume very different values, lead to unbalanced benefit $U_{A}^{\Omega (t)}$ and $U_{B}^{\Omega (t)}$. This is also the case for the random procedure.

Each point in Fig. 4 represents the time average of the differences (absolute values) $\bar{\Delta U\;}$ between the increments to the benefit achieved by each of the two counselors for a selected country, at each time *t*:


(15) $$\bar{\Delta U}\equiv \frac{1}{\tau }\sum_{t=0}^{\tau }\left|U_{A}^{\Omega (t)}-U_{B}^{\Omega (t)}\right|.$$

This is evaluated over the time interval *τ* encompassing the entire vaccination period. The results for the diverse strategies are depicted for each of the selected countries. These suggest that the imbalance $\bar{\Delta U}$ approaches null values through the envy-free strategy. Relatively large values are obtained by applying the other two strategies and also by choosing the regions at random.

Although each of the benefits accumulates to the unity simultaneously, the way this is accomplished and the effects on the achievements of the two counselors can differ considerably. The comparative results in Fig. 5 using data from the United States illustrate the time evolution of the *cumulative benefits*  $\Phi_{\eta }(t)$


(16) $$\Phi_{\eta }(t)=\sum_{t^{´}=0}^{t}U_{\eta }^{\Omega (t^{´})}$$

for each of the two counselors η=A,B, and the mean:


(17) $$\Phi (t)=\frac{1}{2}(\Phi_{A}(t)+\Phi_{B}(t)).$$

These offer information about the efficiency with which the benefits are acquired under different strategies. This can be better seen by interpreting cumulative data as the positions in time of two particles in the space of benefits driven, each of them, by the corresponding counselor. The outcomes obtained for all selected countries exhibited a similar pattern (results not shown).

Figure 6 depicts the time average of the differences (absolute values) between the contributions to the cumulative benefits of the two counselors, evaluated for all the countries listed:


(18) $$\bar{\Delta \Phi }=\frac{1}{\tau }\sum_{t}\left|\Phi_{A}(t)-\Phi_{B}(t)\right|.$$

These represent the average distance kept between the two particles in each case, until reaching their common final position simultaneously. Large values indicate that on average, one of the particles reached positions close to the goal considerably faster than the other. That is, for such strategies, the two benefits evolve out of sync over a considerably large time interval. This is the case of maximize-benefit and oldest-first in these examples. Conversely, the positions of the two particles under the envy-free remain very close together at each instant through the entire time interval, suggesting that in addition to offering a way to promote a balance between acquired benefits, this strategy offers also a way to balance the instantaneous rates at which this happens. This is important for practical purposes since the intervals between consecutive batches may be very large, especially during the initial vaccination periods. The effects of a time delay between the achievements of each of the two benefits may have devastating consequences for the community. Notice also that the random choice procedure offers balanced rates, on average, although the instant rates differ considerably since the sizes of the increments to the benefits alternate unbalanced.

Figure 7 shows the corresponding time average $\bar{\Phi }$ of Φ(t) [17]:


(19) $$\bar{\Phi }=\frac{1}{\tau }\sum_{t}\Phi (t).$$

These include the results for the *minimize-benefit* which is worth considering here precisely because it offers a lower bound to set a scale that allows one comparing outcomes.

Keeping with the kinematic interpretation, the data in Fig. 7 refer to the time averages $\bar{\Phi }$ of the positions of the center of mass of the two particles achieved through the diverse strategies, and for all of the selected countries. The maximize-benefit presents the largest average value, as expected. Even though the partial benefits, i.e. the ones envisaged by each counselor, evolve at different rates in this case, both of them reach large values within relatively short times. Envy-free is also efficient in accumulating benefits almost as fast as the maximize-benefit does. Apparently, in all cases, the oldest-first and the random choice promote the worst results among all the considered procedures, except for a strategy introduced here named minimize-benefit. Under this, one looks for regions of the simplex that minimize the total benefit at each time. Although very implausible to be adopted in practice, this strategy is useful to consider in the present analysis since it provides a lower bound to compare the efficiency of the diverse strategies investigated. Accordingly, the average benefit accumulated upon envy-free is much closer to the quantity accumulated by the maximize-benefit than that accumulated by the minimize-benefit (Fig. 7). Random choice accumulates benefits at an average rate between the maximize and minimize-benefit strategies. The oldest-first spreads its contributions along the interval showing a strong dependence on the population age distribution.

Figure 8 depicts the results for the time averages of the instantaneous difference $\bar{\Delta U}$ (panel a) and also for the time averages of the cumulative difference $\bar{\Delta \Phi }$ (panel b), both against the average cumulative benefit $\bar{\Phi }$. Each point composing each monochromatic set has been achieved using data from the population age distribution of a country (not identified) in a total of 236. The emphasis is given to the different combinations of utilities and strategies employed. In all cases, the results are in line with the behavior depicted in Figs. 4, 6, and 7, for the utility pair named Default. The envy-free is unique in achieving the smallest differences $\bar{\Delta U}$ and $\bar{\Delta \Phi }$ among all strategies and in producing total benefit at a rate that, on average, is the closest to that achieved by maximize-benefit. Although the maximize-benefit (and in parallel, the minimize-benefit) approaches the results for the envy-free regarding the cumulative difference $\bar{\Delta \Phi }$, the dispersion of data is considerably larger in these cases. A surprising outcome from the study in Fig. 8 is that the envy-free reveals a tendency to minimize the dispersion of the distributions for $\bar{\Delta U},\bar{\Delta \Phi }$ and $\bar{\Phi }$ when compared to the corresponding results achieved by the other strategies.

## Discussion and conclusion

The realistic case addressed here is that of deciding about strategies for allocating vaccine doses that become available to a community at a certain frequency but in very small quantities. In the example used, we consider two guidelines focusing on direct and indirect benefits to drive allocation according to a specified dynamic process. We approach this situation by representing each of these guidelines as the priority of a qualified counselor in scoring the entire population ordered by age. Assuming that full protection of an individual is achieved after receiving a single dose, the challenge is to select the group of individuals to be vaccinated every time a new batch becomes available to balance these two contributions. The difficulty relies on the fact that, in general, there is a difference between the amounts a vaccinated individual contributes to each benefit and that this difference may depend on their age. If they contributed equally, any strategy would trivially approach a balanced condition. We claim that the strategy based on an envy-free division for dose allocation optimizes the individual choices of both counselors offering in this way a suitable and efficient choice to achieve such a balance in more general (unpaired) cases. From a technical point of view, our approach adapts the constructive analysis of the classic cake-cutting problem (9) (i) to include the benefits coupled with the other’s choice, (ii) to allocate the doses from each batch accordingly, and (iii) to introduce a dynamic to resume allocation—referred to as the repeating procedure—each time a new batch becomes available.

Collectively, the results shown in the last section indicate that among all of the considered strategies the envy-free promotes a good balance between the benefits envisaged by the two counselors across time, and also that this happens at similar and relatively high rates at initial times resulting in its fast accumulation. In addition, the envy-free is the strategy that tends to equalize benefits, averaged in time, among diverse countries, which is desirable within a scenario of a pandemic. We thus believe that the proposed strategy fulfills the requirements stated in the introduction since it maintains the balance in agreement to different measures comprising (i) the amount of benefit acquired at each time by each counselor, (ii) the efficiency of the process given the speed with which the cumulative benefit approaches limiting values, and (iii) the tendency to equalize the effects achieved by distinct population age distributions.

Figure 9 exhibits the kind of practical outcome provided by the envy-free for each choice of utilities (Table 1) exemplified for the population age distribution of the United States. For a total *V* of doses in each batch, the curves indicate the number of individuals $\mu_{k}^{\mathrm{EF}}(t)$ from each age group *k* to be vaccinated within the intervals between batches to keep the balance between the two guidelines. These results can be used as suggestions for decision-makers under local public policies. In all cases examined, individuals aged 65 + and those within 15–24 years old are indicated to be vaccinated across the initial batches. This result follows roughly the priorities attributed by the counselors in the examples. The difficulty is precisely to figure out the set of numbers $\left\{\mu_{k}^{\mathrm{EF}}(t)\right\}$, or, equivalently, the percentage of the available batch to be allocated to each age group, at each time *t*. In general, these results depend strongly on the choices of utilities. For example, in the unlikely case for which the utilities attributed by both counselors were increasing functions of age, the results from both maximize-benefits and oldest-first strategies would lead to a balanced allocation. Our choices in Table 1 aim to illustrate more general cases for which the utilities are unpaired.

We have designed the numerical code for implementing the diverse strategies by assuming the same number *V* of doses in all batches and the same time interval *T* between batches. This seems unrealistic since neither the time between batches nor the number of doses is predictable in this way, in general. We have also considered that the scores attributed by the counselors to the different age groups do not change across the vaccination periods. We restricted our goals to a comparative analysis of the diverse strategies for a given set of parameters that define *V*, *T*, and the scores giving rise to the utility functions. Nevertheless, the code can be easily adapted to more general situations because these parameters can be chosen differently each time the simplex is rescaled, to resume the next step when new doses are allocated. Concerning this, the envy-free strategy is easily adapted to other scenarios, including, for instance, the ones for which it becomes necessary to account for age-specific seroprevalence, by modifying the utilities accordingly. The model is not in any possible way conditioned to the specific guidelines addressed above. These have been selected as references to explain and illustrate the practice of the method. Any other guideline could have been chosen to drive the allocation. In addition, because Sperner’s Lemma can be extended to more dimensions (9, 10), this opens the possibility to extend the constructive procedure described above to approach more realistic situations for which there are more than two guidelines defining priorities (3).

### Interaction with epidemiological models

Other vaccination schemes have been considered in the literature concerning the time evolution of compartmentalized populations in age-stratified epidemiological models of SIR type. See, for example Refs. (14–17). The study in Ref. (16) is based on input quantities named $\mu_{k}$, which represent the number of individuals in each age group *k* that receive the doses at each instant of time. The time evolution of susceptible, infected, and recuperated (or dead) populations is usually presented for a given set $\left\{\mu_{k}\right\}$ proportional to the susceptible individuals in each group, modeled by certain weighting factors chosen to test ways to control peak infections, peak occupancy in Intensive Care Units (ICU), and deaths related to the disease. This suggests conceiving a vaccination plan based on an interplay between the dynamic of the envy-free and that of the SIR models since the outcomes depicted in Fig. 9 seem to fulfill the requirements for a particular set $\left\{\mu_{k}^{\mathrm{EF}}\right\}$ with the property of balancing the considered benefits. Such an interplay between the two dynamics may reveal their complementary nature in improving the results of both models.

We have assumed that vaccination is the only mechanism by which individuals are removed from the simplex. This adapts to cases for which individuals should be vaccinated regardless of whether they have had previous contact with the agents of the disease. We also do not account for eventual changes in morbidity or in age-related transmission rates across the evolution of the pandemics, meaning that the scores assigned by both counselors are not updated across the time that vaccination takes place. Therefore, the results in Fig. 9 might be improved by the predictions from the SIR or even by employing empirical data about the evolution of transmission and morbidity to modify the scores at appropriate times. In turn, the set $\left\{\mu_{k}^{\mathrm{EF}}\right\}$ may have a considerable impact on the predictions about the fate of the individuals in each compartment of the SIR, under this strategy of vaccination. We believe this offers an alternative that may contribute to the achievements in previous studies, and it is worth considering.

## Supplementary Material

pgae087_Supplementary_Data

## Data Availability

The data and code to reproduce results of this article are publicly available in Ref. (18).

## Preprints

This manuscript was posted on a preprint: 10.48550/arXiv.2306.04582.
