# Supplementary material for: Balancing the benefits of vaccination: An envy-free strategy
Source: PNAS Nexus. 2024 Feb 26;3(3):pgae087. doi: 10.1093/pnasnexus/pgae087 (PMC10923509; doi:10.1093/pnasnexus/pgae087)
Supplement: pgae087_Supplementary_Data [file pgae087_supplementary_data.pdf]

Supplementary Material

Methods

We outline below the algorithm we have developed to find the *envy-free* division for vaccine allocation, given a pair of utility density functions.

The time $t = nT$ of the nth iteration is measured in intervals $T = 1$ between the availability of consecutive vaccine batches with V doses each. A fraction $v(t) = V/N(t)$ from the simplex embracing $N(t) > V$ susceptible individuals at t is selected for vaccination and then, removed. The simplex must then be re-scaled in order to map the remaining $N(t+1) = N(t) - V$ susceptible into the interval $[0, 1]$ to resume the process of vaccination at the time $t+1$. Therefore, each iteration of the simulation comprises three steps: a decision step; a removal step; and a re-scaling step, which are sketched in Figure S1.

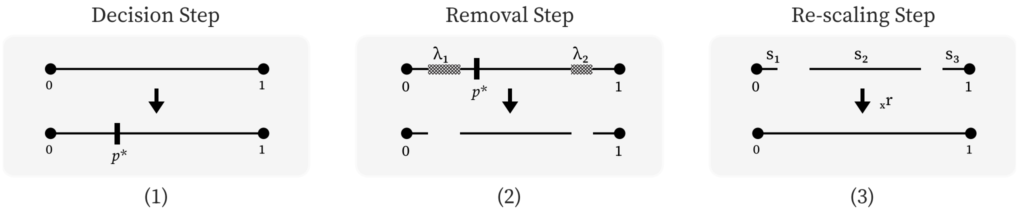

Figure S1: Schematic view of a model simplex at an iteration time t. (1) An *envy-free* division point p^* is identified. (2) The regions λ_1 and λ_2 corresponding to the fractions of individuals that received the doses are removed from the simplex. (3) The remaining regions s_1, s_2, and s_3 are reset through the scale factor r to recompose the simplex for the analysis at time $t+1$.

Decision Step

This is the part that distinguishes the strategies to drive vaccination. To proceed with the *envy-free*, we follow the procedure detailed in Section 2 to build the simplex, label it, and use Equations (6 - 9) to determine which side each counselor would choose to vaccinate at each labeled point. Then, by inspection, we identify all pairs of points (p_L, p_R) between which an *envy-free* point $p^*(t)$ must be located. We choose the average positions between p_L and p_R to approximate the actual $p^*(t)$ at each time. The structure of the simplex with the continuous functions $\rho_\eta(y)$ to approach the utility densities of counselor $\eta = A, B$, guarantees the existence of at least one pair (p_L, p_R) inside the interval.

To build up the functions $\rho_\eta(y)$ we suppose that a value $\psi_k^{(\eta)}$ is attributed by counselor η, as specified in Table 1 (main text), to each age group labeled $k \in \{1, ..., m\}$, that decompose the population of the interval $[0, 1]$ into m sub-intervals, each of these enclosed between initial and final points, respectively y_k^I and y_k^F, for all k. Continuity at the frontiers between neighboring sub-intervals is assured by means of Sigmoid functions with an additional parameter B coinciding with the slope at the origin:

$$G(y) = \frac{1}{1 + e^{-By}}. \tag{S1}$$

This allows a construction of the functions $\rho_\eta(y)$ as

$$\begin{aligned} \rho_\eta(y) = &\psi_1^{(\eta)} \left[1 - G(y - y_1^F)\right] + \\ &\sum_{k=1}^{m-1} \psi_k^{(\eta)} \left[G(y - y_k^I) - G(y - y_k^F)\right] + \psi_m^{(\eta)} \left[G(y - y_m^I)\right] \end{aligned} \tag{S2}$$

The normalized utility density functions $u_\eta(y)$ defined by Equation (1) for each η are evaluated with $\rho_\eta(y)$ defined above.

A remark is in order here regarding an eventual identification of several *envy-free* points in the simplex, at each time t. When this is the case, we proceed by choosing the one leading to the smallest difference between the benefits envisaged by the two counselors. In case of a tie, we choose the *envy-free* point leading to the greatest benefit resulting from adding the two contributions. If the simplex would still present more than one *envy-free* point, we select one of them randomly.

Removal Step

The choice of an *envy-free* point at the Decision Step prescribes a set Λ of $L(t)$ intervals $\Lambda = \{\lambda_1(t), ..., \lambda_L(t)\}$ $\lambda_l(t) \in [0,1]$, each one of them selected either by C_A or by C_B to maximize each one benefit, accounting for the coupled contribution from the other´s choice. The union of all $\lambda_l(t)$, $l \in \{1, ..., L\}$, corresponds to the fraction $v(t) = V/N(t)$ of the population vaccinated at time t which is then removed from the interval $[0,1]$. At the end of this removal process occurring at the interaction time t, the simplex turns out into a set of $L(t)+1$ disjoint intervals $s_j(t) = [s_j^I(t), s_j^F(t)]$, $j \in \{1, .., L(t)+1\}$ which union

$$S(t) = \bigcup_j s_j(t) \subseteq [0,1] \tag{S3}$$

shall be re-scaled to define the simplex at the time $t+1$. The construction is sketched in **Figure S1** for $L = 2$.

Re-scaling Step

After removing the individuals vaccinated at time t, each interval $s_j(t)$ is re-scaled into a new interval referred to as $\zeta_j(t+1)$. The union of all $\zeta_j(t+1)$ defines the new simplex $[0,1]$ over which the former steps shall be repeated at time $t+1$:

$$Z(t+1) = \bigcup_j \zeta_j(t+1) = [0,1] \tag{S4}$$

The intervals $\zeta_j(t+1)$ are set through the scale factor

$$r(t;t+1) = \frac{N(t)}{N(t+1)} = \frac{1}{(1-v(t))} \tag{S5}$$

so that the first interval $\zeta_1(t+1)$ has its endpoints calculated as:

$$\begin{aligned} \zeta_1^I(t+1) &= 0 \\ \zeta_1^F(t+1) &= \Delta s_1(t) r(t;t+1) \end{aligned} \tag{S6}$$

where $\Delta s_j(t) = s_j^F(t) - s_j^I(t)$ is the size of the interval $s_j(t)$. The remaining intervals $[\zeta_j^I(t+1), \, \zeta_j^F(t+1)]$ for all $j > 1$ are set as:

$$\begin{aligned} \zeta_j^I(t+1) &= \zeta_{j-1}^F(t+1) \\ \zeta_j^F(t+1) &= \zeta_j^I(t+1) + \Delta s_j(t) r(t; t+1) \end{aligned} \tag{S7}$$

$Z(t+1)$ (S4) defines the simplex that will be considered in the next iteration, at the time $t+1$. The three steps described above are iterated up to the vaccination is completed.

About the choice of parameter d

Sperner's Lemma guarantees that the *envy-free* strategy will always find at least one pair of points (p_L, p_R) enclosing an *envy-free* point p^* at the end of each iteration time. However, if the considered number d of divisions of the simplex is too small, the average between these two points may not be a good approximation to p^*, as we have assumed. In this case, changing d may lead to oscillations in the value of p^* and, most probably, in the quantities derived from it. Improving the approximation by increasing d is expected to reduce such oscillations as p^* approaches its actual value. This, however, implies adding considerable computational costs to the numerical procedure. To achieve a compromise between mathematical accuracy and computational performance in this case, we examine how the change in d directly affects the temporal averages of the quantities shown in **figures 4, 6, and 7** using as an example, the population age distribution from the U.S.. **Figure S2(a)** shows the average temporal behavior of the difference of the benefits (absolute values) (Equation 15) due to the contributions of the two counselors obtained through the *envy-free* strategy, as d increases. **Figure S2(b)** shows the corresponding behavior of the cumulative differences (Equation 18), and **Figure S2(c)** shows the mean cumulative benefit (Equation 19). As expected, the amounts oscillate around the mean until stabilizing at a certain value of d that is not the same for the different quantities analyzed. We proceed into the whole numerical calculation presented above choosing $d = 100$ which seems suitable to ensure convergence of the results in all cases.

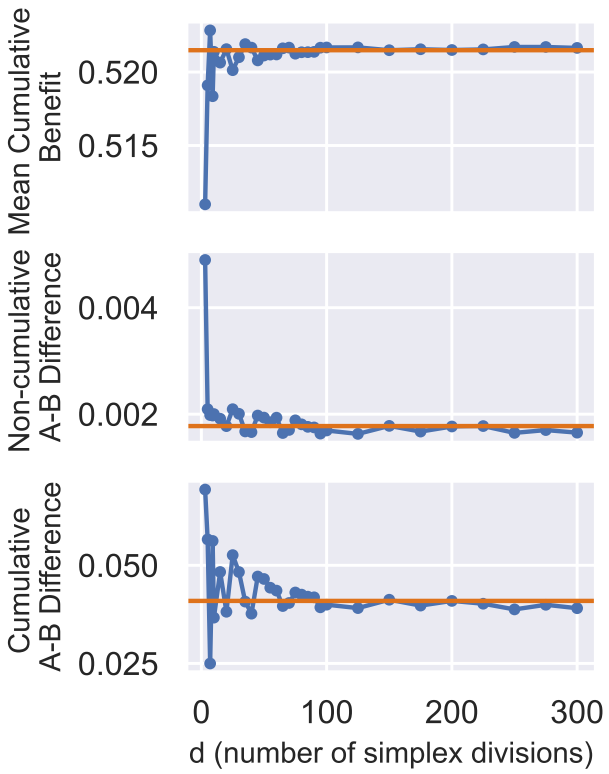

Figure S2: The study of convergence of the results as d varies. The quantities examined are indicated on the axis. In each case, the median is indicated by the orange line.

Other strategies

The other strategies considered to simulate the dynamics of vaccination, in particular the *maximize benefit* and the *oldest-first*, introduce changes into the Decision Step described above.

The *maximize-benefit*, is based on the choice of the region $\Omega(t)_{\max}$ in the simplex which is of the size of the total fraction $v(t) = V/N(t)$ of individuals to be vaccinated at each time t, such that it maximizes the total benefit, accounting for both counselors according to the prescription in (Equation 14) for $\Omega(t) = \Omega(t)_{\max}$. The iterating procedure follows then the same removal and re-scaling steps as for the *envy-free*.

The implementation of the *oldest-first* strategy consists in allocating the total fraction $v(t) = V/N(t)$ of vaccine doses available at the time t to the oldest fraction of the population present at that time. The resulting benefit is evaluated according to (Equation 14) for $\Omega(t) = \Omega(t)_{\text{oldest}}$. The iterating procedure follows then the same removal and re-scaling steps as for the *envy-free*.
